# Identification of the appropriate fixation site to avoid peritoneal catheter migration based on a mechanical analysis

**DOI:** 10.1080/0886022X.2017.1291433

**Published:** 2017-02-22

**Authors:** Yujuan Wang, Yao Zou, Xinghua Chen, Jili Zhu, Cuizhi Xiang, Houjun Jia, Guohua Ding, Huiming Wang

**Affiliations:** a Renal Department of Renmin Hospital, Wuhan University, Wuhan, PR China;; b School of Urban Construction, Wuhan University of Science and Technology, Wuhan, PR China;; c Division of General Surgery, First affiliated hospital of Chongqing Medical University, Chongqing, PR China

**Keywords:** Peritoneal dialysis, abdominal wall fixation, finite element method

## Abstract

**Aim:** To conduct mechanical analysis on the relationship between abdominal wall fixation point and the displacement of catheter top, and establish the finite element model for the complex forces and conditions that the catheter wears in human abdominal cavity, in order to provide the scientific basis for optimizing the catheter position in abdominal wall fixation method.

**Methods:** Using the PIPE59 finite elements to divide units, and taking the lower part of catheter, that is, below interior polyester cuff to simulate and compute the displacement formula.

**Results:** The whole model includes a total of 1701 units. Periodic load was used to simulate the dynamic pressure that peritoneal dialysis catheter gets in abdominal cavity. The load direction was perpendicular to the catheter axis. We used pressure amplitude, duration and frequency as the boundary conditions, and adjusted the fixation point of the catheter lower part at the same time, thus calculating the extreme displacement value of the catheter top end with changing parameter conditions. We also did fitted regression on the results and obtained the displacement formula: *y* = 0.2 × 0.87^x^ (y: the end displacement of peritoneal dialysis catheter, x: the distance between fixation point and the interior polyester cuff), *R*^2^: .982. Simulation the catheter maximal displacement on flat surface demonstrated that additional catheter fixation at the site of 9 cm or more below the internal cuff significantly restricted the catheter migration.

**Conclusions:** The optimal position of fixation point in peritoneal dialysis is about 9 cm away from the interior polyester cuff.

Peritoneal dialysis is one of the primary ways in renal replacement therapy for end-stage renal disease (ESRD). Successful peritoneal dialysis depends on the optimal positioning of peritoneal dialysis catheter and proper handling of complications. An important factor affecting the implementation of peritoneal dialysis is the catheter-related complications, among which the dialysis catheter migration occurs in 10–22% cases^1^ and imposes 22 times higher risk of catheter failure over that caused by infection complications.^2^ It is of great clinical significance to address catheter migration.^3–7^ In general condition, the catheter tip is placed into the Douglas cavity in the catheter insertion, and the inner cuff of the catheter is fixed in the abdominal wall but elsewhere of the intra-abdominal segment of the catheter is not fixed. It can be assumed that the catheter tip may swing to migrate under various forces, that are derived from the strains and stresses of the catheter itself, the gastrointestinal motility, or from patient itself violent coughing and sudden movements. Actually, studies from us and others have proved the necessary of additional catheter fixation to avoid migration.^8–14^ However, the appropriate fixation position is not precisely defined yet. If the fixation position is too proximal to the inner cuff, the catheter is hard to be restricted and the possibility of catheter displacement is still exist. On the other side, if the fixation position is too far from the inner cuff (or near the catheter tip), it increase the operation difficulty and may result in the distal catheter end pressing against the bladder, which can cause pain and discomfort to patients after surgery. Evenmore, the catheter tip may release from the fixing stitch if it is too close to the catheter end. To our knowledge, the appropriate fixation point has not been recommended in the peritoneal catheter insertion, and is usually roughly set relying on experience. In this study, we did mechanical analysis on the relationship between abdominal wall fixation point and the catheter top displacement, in order to provide a scientific computational evidence for peritoneal catheter fixation.

This study involved no human or animal study and is approved by Ethical Committee of Renmin Hospital, Wuhan University.

## 1. Establishing the finite element model

### 1.1. The modeling equipment

Hardware: DELL T7910 tower workstation. Software: large-scale finite element analysis software ANSYS version 13.0 (ANSYS, Inc., Canonsburg, PA); image processing software origin 8.0 (National Institutes of Health, Bethesda, MD).

### 1.2. Modeling process

The cross-section of model used the rings with 3 m thick wall. The upper axis was modeled by a straight line, and the lower axis was simulated by involute.

PIPE59 unit is a single-axis unit that can withstand tensile, compressive and bending forces, as well as simulate the ocean waves and currents. It has good simulation effect for the silicone tube in dialysis liquid. Each node of the unit has six degrees of freedom, which are the linear displacements along x, y, z directions and the angular displacements encircle X, Y, and Z axis. The element force of this unit includes hydrodynamic force and buoyancy action; the element mass includes attached water mass and interior water mass. This unit is very suitable for studying stiffness hardening and nonlinear large strain problems.

**Figure 1.**
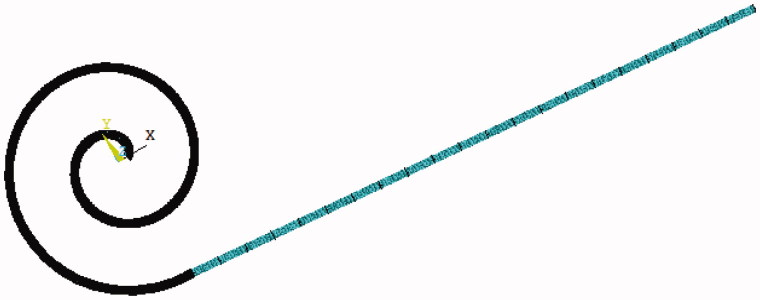
Finite element model.

**Figure 2.**
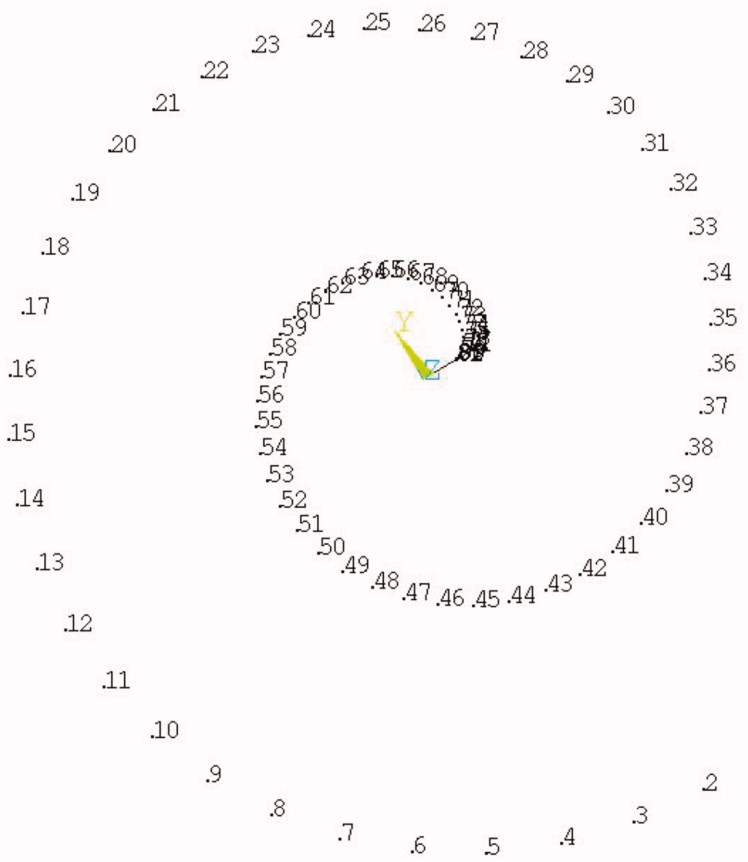
The numbers of involute nodes.

Assume that the interior polyester cuff is the fixation end, and take 20 equidistant points on the upper part of the model, then there are totally 1701 units can be obtained. The assemble model is shown in Figures 1 and 2.

### 1.3. Assumptions and boundary conditions

Strictly speaking, silicone is a typical material, that is, easily deformed, incompressible, nonlinear and hyperelastic. To simplify the calculation and make it easier for the qualitative analysis of this study, we first made the following assumptions: ① small deformation assumption: when the strain is less than 10%, the silicone material is isotropic; ② the analysis within the plane: if the silicone tube is deformed within the plane, then the displacement outside of the plane can be ignored. The density of the silicone tube is 1.1 × 103 kg/m^3^, the elasticity modulus is 2.14 MPa, and the Poisson’s ratio is 0.48.

### 1.4. Load case

Based on clinical experience, it can be assumed that the pulsating load cycle in abdominal cavity is 0.25 s, and the abdominal velocity amplitude is 0.05 m/s. Suppose that the pulsating pressure distributes from 0 to 360 degrees, then each lower end fixation point corresponds to 24 working conditions with a 15 degree interval, thus there’s a total of 20 × 24 = 480 conditions. From each condition, we took the maximum displacement value of the dialysis catheter’s bending top as the calculation result.

## 2. Results analysis

**Figure 3.**
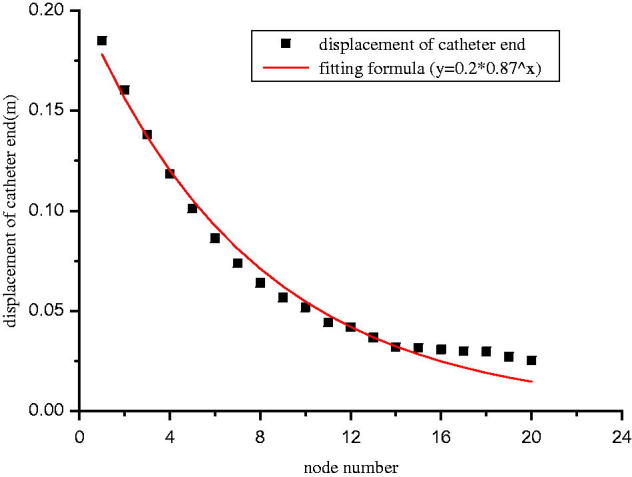
Calculation result and fitting formula.

We used periodical load to simulate the dynamic pressure that peritoneal dialysis catheter got in abdominal cavity. The load direction is perpendicular to the axis of catheter, and the pressure amplitude, duration, frequency were taken as boundary conditions. We adjusted the position of lower catheter fixation point, while calculating the spatial extreme displacement value of the catheter top end with changing parameter conditions. We analyzed the results and obtained the fitted regression equation for displacement (see Figure 3): *y* = 0.2 × 0.87^x^ (y: the top end displacement of catheter, x: the distance between fixation point and the interior polyester cuff), *R*^2^: .982. The relationship between fixation point and catheter displacement is shown in Table 1.

**Table 1.** The relationship between fixation point and catheter displacement.

*N*	1	2	3	4	5	6	7	8	9	10
L	0.65	1.30	1.95	2.60	3.25	3.90	4.55	5.20	5.85	6.50
Δ	18.50	16.03	13.81	11.84	10.11	8.63	7.40	6.41	5.67	5.18
*N*	11	12	13	14	15	16	17	18	19	20
L	7.15	7.80	8.45	9.10	9.75	10.40	11.05	11.70	12.35	13.00
Δ	4.44	4.19	3.68	3.21	3.17	3.08	3.00	2.98	2.72	2.54

*N* is the node number, L is the distance between fixation point and the interior polyester cuff (cm), and Δ is the catheter top displacement (cm) calculated from the finite element model.

## 3. Test the effects of catheter fixation site on the possible catheter maximal displacement distance on flat surface

**Figure 4.**
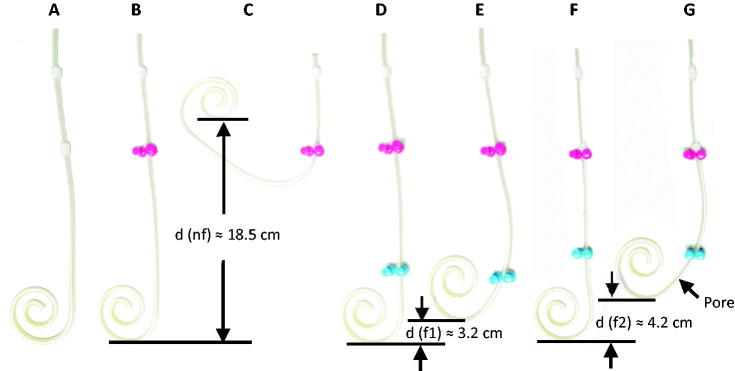
Simulation the maximal catheter displacement distance and the fixation site on a flat surface.

To testify the effects of catheter fixation site on the possible catheter maximal displacement distance, the standard tenckhoff curled catheter was placed on a flat surface (Figure 4(A)). The internal cuff was fixed on the surface to simulate the state of catheter being inserted into the peritoneal cavity (Figure 4(B)). In this condition, the complexity of the forces acting on the catheter in real peritoneal cavity was simplified by the vertical force constitution of gravity downward and flat prop up. To simulate the catheter displacement, an assumed force along horizontal plane was added to force the catheter bend upward to the maximal degree, until at which the catheter stay immobile under the actions of opposite forces of mechanical stress from catheter deformation and the friction force from the flat surface. The displacement distance of the catheter curl bottom was then measured (Figure 4(C)). The catheter was fixed additional on the surface at the site 9 cm (Figure 4(D,E)) or 8 cm (Figure 4(F,G)) far away from internal cuff, respectively, the maximal catheter displacement distance was measured similarly. It demonstrated that the maximal catheter displacement distance reached to approximate 18.5 cm, 4.2 cm, and 3.2 cm in the conditions of no additional catheter fixation, additional fixation in the site of 8 cm, or 9 cm below the internal cuff of the catheter. This suggested that the maximal catheter displacement would be very slight if additional catheter fixation is performed at the site of 9 cm or more below the internal cuff.

## 4. Discussion

The occurrence of dialysis catheter migration is associated with various factors, including severe peristalsis (diarrhea), improper sleep posture, intense exercise, different catheterization methods, etc. These factors can all lead to catheter end bending upward and the whole tube shift. First, the peritoneal dialysis catheter is made of silicone which has lower density. Therefore, when the catheter is placed in peritoneal dialysis fluid that has higher proportion, an upward buoyancy will act on the catheter. Especially, when the patient is in the supine position, majority of the catheter is placed in peritoneal dialysis fluid, and the buoyancy is close to gravity. So, it is more likely for the catheter drift to happen when the patient is in supine position than in sitting or standing position. Secondly, the catheter position is affected by gastrointestinal peristalsis. Constipation, diarrhea, or other abnormal peristalsis, as well as increased abdominal pressure, are all incentives for catheter migration. When intestine peristalsis slows down, the holding effect of descending colon’s downward movement that can keep the lowest position of catheter is reduced, which is one of the reasons that elderly patients and diabetic patients are more prone to have drift catheter. In addition, repeated pulling of the catheter can cause the constraint force of catheter’s abdominal wall segment to change its direction. Thereby catheter drift can also occur due to the torsion from catheter fixation end. Huiming Wang et al.^15^ suggested that the catheter can be subjected to the action of three forces in abdominal cavity: the traction force from surrounded omentum majus, buoyancy, and the dynamic force from abdominal peristalsis. The combined action of these forces can cause catheter migration, affecting the process of peritoneal dialysis. In order to reduce the incidence of catheter migration, both domestic and foreign clinicians added the abdominal wall fixation method onto the traditional surgical method, but the fixation method is not firmly defined yet. If the fixation point is too shallow, then the catheter cannot be effectively fixed; if the fixation point is too deep, then it may result in the distal catheter end pressing against the bladder, which may cause pain and discomfort to patients after surgery. Huiming Wang et al.^14^ chose the position at 10–14 cm below the belly button as the fixation point, and used the Wang’s-forceps to fix the catheter lower part. Jinari et al.^16^ fixed the catheter at 2 cm below the upper incision and 2–3 cm under the pouch. The fixation site that Guoqin Lu et al.^17^ chose was 7–9 cm below the catheter incision. Yu Tong et al.^18^ conducted the incision and fixation at the position about 2.5 cm below the pouch. Therefore, the selection of fixation point is more relied on the clinical experience of researchers, lacking the scientific evidence to serve as a theoretical basis.

The finite element method is a numerical method for solving the mathematical physics problems in engineering. The basic idea of this method is to discretize the solution domain into a set of unit combinations that have finite number and are mutually coupled in a certain way. Each unit can be combined with other unit in different kinds of connections. Therefore, this method can simulate the solution domain with complex geometry. Human organs, tissues, and some medical devices all have irregular geometrical shapes. With the finite element method, people can establish the finite element model after discretize and compose the objects, and are able to conduct a variety of mechanical analyses without having to establish an *in vitro* physical model. In recent years, the finite element method already had many applications in medical field, including biological simulation experiments, finite element modeling, mechanical property evaluation of medical devices, and optimization of the device design, etc. The spinal biomechanics simulation is the field where the finite element method was applied the earliest, most, and most widely. Be-lytschko et al.^19^ first applied the finite element method in biomechanics research, and established a two-dimensional intervertebral disc model, which marked the beginning of the applications of finite element method in orthopedic biomechanics research. After that, Liu et al.^20^ first proposed the three-dimensional finite element model, expanding the applications of finite element method in thoracolumbar research. Recently, with the advances in digital and computational technology, finite element method is more and more involved in establishing the dynamics models of various human organs and medical equipment, including eyes,^21^ teeth and orthotic devices, stomach, joints,^22^ etc., which played an important role in clinical radiology, physical measurements, parameter optimization and selection. So far, finite element method has become a key tool in biomechanical study.

Therefore, we based on the nonlinearity of catheter’s material and geometry, as well as the theoretical tools, such as structural dynamics, hydromechanics, random vibration and mathematical statistics, used the existing mature finite element software, and measured the force boundary conditions of catheter in live animal model. Then, we established the refined dialysis catheter model, and hoped to be able to analyze and obtain the optimal solution for catheter fixation pattern.

In this study, we established the finite element model of catheter, adjusted the lower catheter fixation positions, calculated the spatial extreme displacement value of catheter top end with changing parameter conditions, conducted fitting regression on the results, and obtained the displacement formula: *y* = 0.2 × 0.87^x^. With the fixation point going down, the catheter top displacement gradually decreased. Therefore, the movement of catheter end is limited after adding the abdominal wall fixation method onto peritoneal dialysis catheter, which effectively decreases the incidence of catheter migration. However, in the actual clinical cases, it is not always better when the fixation point goes further away from the interior polyester cuff. If the fixation point is too deep, it may result in the distal catheter end pressing against the bladder, which can cause pain and discomfort to patients after surgery, and the surgery is more difficult to operate. To find the best fixation point, we evenly divided the straight catheter segment below the polyester cuff into 20 nodes, with a 0.65 cm interval. Then, we assumed the fixation point at each node in the order from the 1st to the 20th, and calculated the catheter displacement according to the formula. When the fixation point moved between the 1st and the 14th node, the catheter displacement significantly decreased with the fixation point going down; but when the fixation point continued to move down to the 15–20th node, the displacement showed very small changes. So the position of the 14th node, which is about 9 cm away from the interior polyester cuff, was considered to be the best position to do catheter fixation. Therefore, without considering the individual differences between patients, the results suggested that the proper position of dialysis fixation point is about 9 cm away from the interior polyester cuff.

This article discussed the principles and mechanisms of abdominal wall fixation from the angle of mechanics numerical simulation, and improved the fixation method based on these principles. It created a theoretical basis for future studies on peritoneal dialysis cathetering, and provided scientific support for the selection of catheter fixation point in clinical practice.
